# BID Mediates Oxygen-Glucose Deprivation-Induced Neuronal Injury in Organotypic Hippocampal Slice Cultures and Modulates Tissue Inflammation in a Transient Focal Cerebral Ischemia Model without Changing Lesion Volume

**DOI:** 10.3389/fncel.2016.00014

**Published:** 2016-02-03

**Authors:** Nellie Anne Martin, Helena Bonner, Maria Louise Elkjær, Beatrice D’Orsi, Gang Chen, Hans Georg König, Martina Svensson, Tomas Deierborg, Shona Pfeiffer, Jochen H. Prehn, Kate Lykke Lambertsen

**Affiliations:** ^1^Department of Neurology, Institute of Clinical Research, Odense University HospitalOdense, Denmark; ^2^Department of Physiology and Medical Physics, Centre for the Study of Neurological Disorders and 3U-COEN, Royal College of Surgeons in IrelandDublin, Ireland; ^3^Department of Neurobiology Research, Institute of Molecular Medicine, University of Southern DenmarkOdense, Denmark; ^4^Department of Experimental Medical Sciences, Experimental Neuroinflammation Laboratory, Lund UniversityLund, Sweden

**Keywords:** BID, focal cerebral ischemia, organotypic hippocampal slice cultures, neuronal injury, inflammation

## Abstract

The BH3 interacting-domain death agonist (BID) is a pro-apoptotic protein involved in death receptor-induced and mitochondria-mediated apoptosis. Recently, it has also been suggested that BID is involved in the regulation of inflammatory responses in the central nervous system. We found that BID deficiency protected organotypic hippocampal slice cultures *in vitro* from neuronal injury induced by oxygen-glucose deprivation. *In vivo*, BID-knockout (KO) mice and wild type (WT) mice were subjected to 60 min of transient middle cerebral artery occlusion (tMCAO) to induce focal cerebral ischemia, and allowed to recover for 24 h. Infarct volumes and functional outcome were assessed and the inflammatory response was evaluated using immunofluorescence, Western blotting, quantitative PCR (qPCR) and Mesoscale multiplex analysis. We observed no difference in the infarct volume or neurological outcome between BID-KO and WT mice. The inflammatory response was reduced by BID deficiency as indicated by a change in microglial/leukocyte response. In conclusion, our data suggest that BID deficiency is neuroprotective in an *in vitro* model and modulates the inflammatory response to focal cerebral ischemia *in vivo*. However, this is not translated into a robust neuroprotection *in vivo*.

## Introduction

Within minutes following the incidence of an ischemic stroke, necrotic cell death is initiated in the core region of the infarct. In the ischemic penumbra, nutrient deprivation is less severe and the damage is transiently reversible. Here, cells often die from apoptosis initiated hours after the onset of ischemia in a process that may last for days (Astrup et al., 1981; Zivin, 1998; Mergenthaler et al., 2004; Lorz and Mehmet, 2009). The BH3 interacting-domain death agonist (BID) is a proapoptotic B-cell lymphoma-2 (BCL-2) family protein involved in death receptor-induced apoptosis. BID links death receptor signaling to the mitochondrial BCL-2 family-controlled apoptotic pathway (Wang et al., 1996; Engel et al., 2011). The involvement of BID in apoptosis is believed to be stimulated by cleavage of full length-BID in the cytosol to form truncated BID (tBID). This leads to translocation of tBID to the outer mitochondrial membrane resulting in cytochrome c release and thereby apoptosis (Broughton et al., 2009; Ren et al., 2010; Engel et al., 2011).

BID has been considered a significant player in cell death following focal cerebral ischemia, as BID-knockout (KO) mice developed smaller infarcts than wild type (WT) mice (Plesnila et al., 2001; Yin et al., 2002). In addition to apoptosis, it has been suggested that BID is also involved in inflammatory signal transduction in a process independent of its proapoptotic properties. This has been tested *in vitro* using BID-deficient microglia, astrocytes, macrophages and human colon adenocarcinoma (HT29) cells, and in serum from mice exposed to intraperitoneal injections of the bacterial peptide L-Ala-γ-D-Glu-*meso*-diaminopimelic acid (γTri-DAP; Mayo et al., 2011; Yeretssian et al., 2011; König et al., 2014). In these studies BID deficiency resulted in decreased levels of proinflammatory cytokines and chemokines (Mayo et al., 2011; Yeretssian et al., 2011). Furthermore, it has been described that BID deficiency is associated with lower levels of tumor necrosis factor (TNF), interleukin (IL)-6 and chemokine (C-C motif) ligand 2 (CCL-2) in a disease model for sepsis (Chung et al., 2010) and with higher levels of IL-1β and chemokine (C-X-C motif) ligand 1 (CXCL1) in experimental inflammatory arthritis (Scatizzi et al., 2007). In addition to altering the expression pattern of cytokines and chemokines, BID deficiency has also been shown to reduce neutrophil infiltration following experimental peritonitis in mice (Yeretssian et al., 2011), and to impair phagocytic properties of microglia and macrophages (Mayo et al., 2011). Mechanistically, it has been proposed that the observed effects of BID deficiency on inflammation is attributed to the observation that full-length BID is required for nucleotide-binding oligomerization domain containing (NOD) 1 and NOD2 signaling thereby influencing extracellular signal-regulated kinase (ERK) and nuclear factor κB (NF-κB) signaling (Yeretssian et al., 2011). However, this hypothesis has been questioned (Mayo et al., 2011; Nachbur et al., 2012). In the present study, we examined the effect of BID deficiency in an *in vitro* model of ischemic neuronal injury, and examined the effect of BID deficiency on lesion size, functional outcome and inflammation in response to focal cerebral ischemia in mice.

## Materials and Methods

### Mice

BID-KO mice (Kaufmann et al., 2007) were provided by Prof. Andreas Strasser, WEHI, Melbourne, Australia and have previously been described (Kaufmann et al., 2007; König et al., 2014). The BID-KO mice were generated on an inbred C57BL/6 background, using C57BL/6-derived embryonic stem cells. Corresponding C57BL/6 WT mice were from the same background. For focal ischemia experiments, male mice aged 9–11 weeks weighing 25–28 g were used. Mice were housed in numbers of five per cage under diurnal lighting conditions, allowed free access to food and water, and cages were provided with shelter, and bedding and nesting material. All animal work was performed with ethics approval and under licenses granted by the Irish Department of Health and Children. Animal procedures were reviewed and approved by the RCSI Research Ethics Committee.

### DNA Extraction and Genotyping

Mice tail DNA was extracted using High Pure PCR Template Preparation Kit (Roche, UK). Genotyping was performed using specific primers as follows: 5′-GGT-CTGTGTGGAGAGCAAAC-3′ (common), 5′-TCAGGTGCCAGTGGAGATGAACTC-3′ (wild type allele-specific) and 5′-GAGTCATACTTACTTCCTCCGAC-3′ (mutant allele-specific) for BID.

### Preparation of Organotypic Hippocampal Slice Cultures

Organotypic hippocampal slices cultures (OHSCs) were prepared according to a previously described procedure (Stoppini et al., 1991; Kristensen et al., 2001; Bonner et al., 2010) with minor modifications. The brains from postnatal day 10 WT and BID-KO mouse pups were isolated and transferred to dissection medium containing HBSS (Invitrogen), 20 mM HEPES, 100 U/ml pen/strep, and 6.5 mg/ml D-glucose (Sigma Aldrich, Ireland). Brains were dissected in half and each hemisphere was separated to expose the hippocampi, which were separated from the neighboring thalamus and basal ganglia and the septo-hippocampal connection severed with a scalpel. Isolated hippocampi were placed on a McIlwain tissue chopper (Mickle Laboratory Engineering, UK), aligned perpendicularly to the chopper blade and cut into 450 μm thick sections. The slices were transferred into fresh dissection medium and selected for clear hippocampal morphology (intact CA regions and dentate gyrus) and placed on the porous (0.4 μm) membrane of Millicell inserts (Merck Millipore, USA). The inserts were placed in six-well tissue culture plates with 1 ml of culture medium consisting of MEM supplemented with 25% horse serum, 4 mM L-glutamine, 6 mg/ml D-glucose, 2% B27 and 50 U/ml pen/strep. The slices were maintained in a humidified incubator with 5% CO_2_ at 35°C with media changes every second day. All experiments were performed at DIV10.

### Oxygen–Glucose Deprivation (OGD) in OHSCs

Slices were tested for viability with Propidium Iodide (PI) added to the medium at a final concentration of 5 μg/ml for 15 min before the OGD experiments. Healthy slices from WT and BID-KO mice were transferred to a hypoxic chamber (COY Lab Products, USA). The hypoxic chamber had an atmosphere comprising 1.5% O_2_, 5% CO_2_, and 85% N_2_, and the temperature was maintained at 35°C. The slices were transferred to wells containing preequilibrated and deoxygenated OGD medium (bubbled with N_2_ for 1 h before use). The OGD medium consisted of the following: 2 mM CaCl_2_, 125 mM NaCl, 25 mM NaHCO_3_, 2.5 mM KCl, 1.25 mM NaH_2_PO_4_, 2 mM MgSO_4_ and mM 10 sucrose, pH 6.8. After 180 min of OGD, the slices were transferred to fresh oxygenated culture medium and placed in normoxic conditions (21% O_2_ and 5% CO_2_), and PI uptake was observed following a 24 h period. sham slices were free from OGD. For experiments involving the use of NMDA antagonist, MK-801, (10 μM/L) was added pre, during and post OGD treatment.

### PI Staining and Quantification of Injury within the OHSC

Neuronal injury was assessed by means of PI staining as previously described (Bonner et al., 2010). Fluorescence images were acquired with an Eclipse TE 300 inverted microscope (Nikon, Japan) and a 4× dry objective with Wasabi Software version 5.0 (Hamamatsu Photonics, Germany). Illumination and exposure (camera and exposure time) were kept constant throughout each series of recordings and between experiments. The CA1 region was selected from WT and BID-KO hippocampal slices after OGD treatment, and the mean fluorescent intensity was determined. The regional gray-level intensity was measured using the Wasabi interactive tool, background gray-level was accounted for and neuronal injury was expressed as a percentage of total injury (0.5 mM NMDA treatment for 1 h and recovery for 24 h). Data were arranged from at least three similarly treated slices and at least three independent experiments were carried out for each condition.

### Induction of Transient Middle Cerebral Artery Occlusion (tMCAO)

Focal cerebral ischemia was induced by transient middle cerebral artery occlusion (tMCAO) as previously described (D’Orsi et al., 2015). Briefly, mice were anesthetized with a mixture of 62% N_2_O, 33% O_2_ and 5% isoflurane (Abbott, Ireland) for 45 s and maintained with 65% N_2_O, 33% O_2_ and 2% isoflurane. Body temperature was maintained at ~37°C using a feedback-controlled heating pad connected to a rectal probe (Heater Control Module; FHC). The eyes were protected by ointment (Bayer, Germany) and an analgesic (Buprenorphine [0.05 mg/kg], Reckitt, UK) was administrated subcutaneously. A flexible laser-Doppler probe (5001 Master, Sweden) was glued onto the exposed left parietal skull over the middle cerebral artery (MCA) territory for continuous monitoring of regional cerebral blood flow (rCBF). A silicone-coated 8-0 nylon monofilament was introduced through the left common carotid artery and advanced carefully into the internal carotid artery up to the level of the MCA where a drop in rCBF to less than 20% of baseline indicated a successful occlusion of the MCA. Wounds were sutured, the laser-Doppler probe was removed, and mice were kept at 34°C and allowed to wake up. Sixty minutes after MCAO, mice were shortly re-anesthetized and ischemia was terminated by removal of the silicone-coated filament. This surgical procedure has been thoroughly evaluated in our laboratory to confirm reperfusion after filament removal.

For postsurgical pain treatment, analgesic was administered every 8 h after surgery for the first 24 h. Sham mice underwent the same procedure except for the actual occlusion of the MCA.

### Group Size and Study Design of *In Vivo* Experiments

The size of the ischemic infarct was measured in a study in which BID-KO (*n* = 8) and WT (*n* = 8) mice were allowed to survive for 24 h after 60 min of tMCAO. In order to evaluate the effect of ischemia on inflammation BID-KO (*n* = 3) and WT (*n* = 3) mice subjected to sham surgery were included. In the analyses of the cerebrovascular anatomy a total of 7 BID-KO and 8 WT mice were included. The strain of the individual mice was blinded to the person performing surgeries and the following analyses. Three mice were excluded in the analyses due to insufficient infarct formation or experimental flaws. No mice died during the experiment.

### Behavioral Analyses

Behavioral tests were performed in a blinded fashion prior to and after tMCAO in order to evaluate functional outcome 24 h after tMCAO.

#### Neurodeficit Score

To evaluate motor function of mice exposed to tMCAO, a scoring system from 0–4 was applied (Nagayama et al., 1999; McCullough et al., 2005) where 0: no observed deficits; 1: forelimb weakness and torso turning to the ipsilateral side when held by tail, but no circling; 1.5: both circling and non-circling walking pattern observed; 2: circling, no straight walking; 3: leaning to the contralateral side at rest; 4: no spontaneous motor activity or barrel rolling. All mice were scored just before reperfusion and again 24 h after reperfusion.

#### Vertical Rope Test

To objectively evaluate the strength of the mice, a vertical rope test was included (modified from Kashiwabuchi et al., 1995 and Brooks and Dunnett, 2009). A 1 cm thick rope was hanging vertically 20 cm from the ground where an open cage containing bedding material was placed. The rope was covered with a plastic tube, which prevented the mice from climbing, except from the lowest 6 cm which where covered by surgical tape allowing the mice to hold on. The mice were held by their tail and the rope was tilted slightly towards the mice to make sure they grabbed the rope with all four limbs positioned head up. The tail and the rope were carefully released, and the time the mice spent on the rope was measured in four trails with at least 5 min in between. Mice were tested 24 h before and 24 h after tMCAO. The trial was included if the mouse stayed more than 3 s and less than 120 s on the rope, and the average time mice spent on the rope was used in the analysis.

### Tissue Preparation

Mice were killed by an overdose of Pentobarbital sodium (Dolethal, LURE Cedex, France) followed by 25 ml saline perfusion. Brains were immediately removed, snap frozen in dry ice cooled isopentane and stored at −80°C until further processing. Fresh frozen brains were cut coronally in five parallel series on a cryostat (Leica CM 1950, Germany). Three of the series were cut into 10 μm thick sections and collected on Superfrost Plus slides (Thermo Scientific, Germany) with 0.5 mm between each section for infarct volume estimations and immunohistochemistry. The rest of the brain was cut into 50 μm thick sections and collected in Eppendorf tubes for quantitative PCR (qPCR), Western blotting and mesoscale multiplex analysis.

For the analyses of the cerebrovascular anatomy, saline perfusion was followed by perfusion with 25 ml of 4% paraformaldehyde (PFA) and then by 0.8 ml of cresyl violet acetate solution (Sigma Life Science, MA, USA). Brains were stored in 4% PFA until further processed.

#### Infarct Volume Estimations

Brain sections were stained with cresyl violet acetate (Sigma Life Science, MA, USA) and microphotographs were acquired using a 1.25 magnification lens (Leica DM 4000 B, Germany). The area of the infarct, the area of the noninfarcted ipsilateral hemisphere and the area of the contralateral hemisphere were measured using the software Leica Application Suite (LAS V3.7), and the direct and indirect infarct volumes and brain sizes were estimated according to the Cavalieri principle and as previously described (Gröger et al., 2005). In addition, a rostrocaudal infarct distribution analysis was performed comparing the infarct area every 1 mm from 2–10 mm from the frontal pole.

#### Immunofluorescence

Air-dried tissue slides were fixed in 4% formalin, permeabilized in 3% Triton X-100 and blocked in 5% donkey serum. Sections were then incubated at 4°C overnight (ON) in blocking media with rabbit anti-ionized calcium-binding adaptor molecule 1 (IBA1; WAKO, VA, USA, 1:500) and mouse anti-glial fibrillary acidic protein (GFAP; Life Technologies, CA, USA, 1:500). After rinsing the slides in 0.1% Triton X-100 they were incubated in secondary Alexa-488-conjugated rabbit anti-goat IgG (Life Technologies, CA, USA, 1:500) for 1 h at room temperature (RT), thoroughly rinsed in 0.1% Triton X-100 followed by incubation with Alexa-568-conjugated goat anti-mouse IgG (Life Technologies, CA, USA, 1:500) 1 h at RT. Hoechst staining was added (1:1000) for 10 min and sections were mounted in FlourSave (Merck Millipore, USA). Digital images were acquired on a fluorescent microscope (Leica DM 4000 B, Germany) at 20× magnification from seven randomly chosen locations surrounding the infarct in border zone normal appearing ipsilateral cortex and corresponding locations on the contralateral hemisphere. GFAP and IBA1 positive cells were counted and the average number of cells (cells per field of view) was used in the analyses.

#### Evaluation of Mouse Cerebrovascular Anatomy

The cerebrovascular anatomy was analyzed in order to compare the posterior communicating arteries (PcomAs) and the MCA territory between BID-KO mice and WT mice. After cresyl violet perfusion brains were removed and the circle of Willis was exposed by carefully pulling down the cerebellum. Superiorly and inferiorly orientated pictures were acquired on a camera connected to a surgical microscope (Leica DFC29, Germany). The diameters of the PcomAs were measured at the widest point and the diameter of the basilar artery (BA) was measured proximal to the posterior cerebral arteries. The diameter of the PcomAs as a percentage of the diameter of the BA was calculated and used in the analysis (Kitagawa et al., 1998). The MCA territory was examined by measuring the distance from the peripheral anastomoses between the anterior cerebral artery (ACA) and the MCA at 2, 4 and 6 mm from the frontal pole (Maeda et al., 1999).

#### Western Blotting

Western blotting was performed on one series of whole brain lysate by extracting proteins in a RIPA lysis buffer (Merck Millipore, Denmark) containing a protease inhibitor cocktail (Roche Diagnostics, Denmark), and protein concentrations were estimated by the Bradford protein assay (Biorad, CA, USA) as described previously (Lambertsen et al., 2009).

Proteins were resolved by SDS-PAGE on 4–12% Bis-Tris gels (Nupage^TM^, Invitrogen, Denmark) and transferred to polyvinylidene difluoride (PVDF) membranes. SeeBlue Plus2 prestained standard (LC5925 Invitrogen, Denmark) was used as molecular weight marker. Following blocking in 5% non-fat dry milk plus 0.05% Tween-20, membranes were incubated ON at 4°C with rabbit anti-BID (Enzo Life Sciences, USA, 1:1000), rabbit anti-GFAP (DAKO, Denmark, 1:1000), rabbit anti-pERK1/2 (Cell Signaling, 1:2000), rabbit anti-pJNK1/2 (Cell Signaling, 1:1000), mouse anti-α-actin (Chemicon, USA, 1:8000), rabbit anti-pIKKα/β (Cell Signaling, 1:1000), rabbit anti-pp38 (cell signaling, 1:1000) or mouse anti-transcription initiation factor IIB (TFIIB; Cell Signaling, 1:1000) in blocking solutions. After thorough wash in TBS-0.05% Tween20, membranes were incubated in horseradish peroxidase (HRP)-conjugated goat anti-rabbit IgG (Cell Signaling, 1:1000) or HRP-conjugated rabbit anti-mouse IgG (DAKO, 1:2000) for 1 h at RT. For detection of HRP activity, a luminol-based enhanced chemiluminescence (ECL) substrate (Thermo scientific, MA, USA) was used and light emission was captured on a charge-coupled device (CCD) camera (2200 PRO Gel Logic Carestream, USA). Mice included in the Western blot analysis for GFAP were selected based on their infarct size choosing the mice with infarct volumes close to the estimated mean infarct volume within each treatment group. Densitometry of Western blots was performed using Image J analysis software (NIH) in accordance with the Image J developers guidelines and data are presented relative to the level of α-actin or TFIIB.

#### Mesoscale Multiplex Analysis

Whole brain pooled protein samples (23–28 mg/ml) from BID-KO and WT mice exposed to tMCAO (pool from five mice (BID-KO) and six mice (WT)) and BID-KO and WT mice subjected to sham surgery (pool from three mice in each group) were applied and protein extractions were prepared as described for Western blotting. To measure cytokine protein levels by the MSD electrochemiluminescence proinflammatory mouse V-Plex Plus Kit (IFNγ, IL-1β, IL-2, IL-4, IL-5, IL-6, IL-10, IL-12p70, CXCL1, TNF; K15012C, Mesoscale) we used a SECTOR Imager 6000 (MSD Mesoscale Discovery, USA) Plate Reader according to the manufacturer’s instructions. Samples were diluted twofold in Diluent 41 prior to measurement and 25 μl was loaded in each well. Data was analyzed using MSD Discovery Workbench software.

#### Quantitative PCR Analysis

qPCR was performed to examine and compare the relative mRNA expression of BAK, BAX, Bcl2, Bclxl, CD11b, CD38, CXCL1, IL-1β, IL-6, IL-10 and TNF between BID-KO mice and WT mice as described previously (Clausen et al., 2005, 2014). Total RNA was extracted using RNeasy mini kit (QIAGEN, Netherlands) and cDNA was synthesized using 2 μg of total RNA and reagents from a High-Capacity cDNA Reverse Transcription Kit (Life technology, CA, USA) according to the manufacturer’s instructions. qPCR analyses were performed using the following conditions: 2 min at 50°C, 10 min at 95°C, 45 cycles of 15 s at 95°C followed by 1 min at annealing temperature, 15 s at 95°C, 3 min at 60°C and finally 15 s at 95°C. TaqMan chemistry was applied for all analyses with the exception of BAK for which SYBRgreen chemistry was applied. Primer sequences can be found in Table 1. Expression results were reported relative to the geometric mean of the expression of the housekeeping genes glyceraldehyde 3-phosphate dehydrogenase (GAPDH) and hypoxanthine phosphoribosyltransferase 1 (HPRT1).

**Table 1:** **Primers and probes applied in qPCR analyses**.

Primer/probe	Sequence	Cat.no./Supplier
BAK F′	5′-CCTTCTGAACAGCAGGTTGC-3′	TagCopenhagen
BAK R′	5′-GACCCACCTGACCCAA-3′	TagCopenhagen
BAX TaqMan assay	Not available	Mm00432051_m1, Life Technologies
Bcl2 TaqMan assay	Not available	Mm00477631_m1, Life Technologies
Bclxl TaqMan assay	Not available	Mm00437783_m1, Life Technologies
CD11b F′	5′-CGGAAAGTGTGAGAGAACTGTTTC-3′	TagCopenhagen
CD11b R′	5′-CTTATAATCCAAGGGATCACCGAATTT-3′	TagCopenhagen
CD11b probe	5′-TCTGTGATGACAACTAGGATCTTCGCAGC-3′	TagCopenhagen
CD38 Taqman assay	Not available	Mm00483146_m1, Life Technologies
CXCL1 F′	5′-GCTGGGATTCACCTCAAGAAC-3′	TagCopenhagen
CXCL1 R′	5′-TGTGGCTATGACTTCGGT-3′	TagCopenhagen
GAPDH F′	5′-TGTCAAGCTCATTTCCTGGTATGA-3′	TagCopenhagen
GAPDH R′	5′-CTTACTCCTTGGAGGCCATGTAG-3′	TagCopenhagen
GAPDH probe	5′-TCCACCACCCTGTTGCTGTAGCCG-3′	TagCopenhagen
HPRT1 F′	5′-GTTAAGCAGTACAGCCCCAAAATG-3′	TagCopenhagen
HPRT1 R′	5′-AAATCCAACAAAGTCTGGCCTGTA-3′	TagCopenhagen
HPRT1 probe	5′-AGCTTGCTGGTGAAAAGGACCTCTCGAAGT-3′	TagCopenhagen
IL-1β F′	5′-CTTGGGCCTCAAAGGAAAGAA-3′	TagCopenhagen
IL-1β R′	5′-AAGACAAACCGTTTTTCCATCTTC-3′	TagCopenhagen
IL-1β Probe	5′-AGCTGGAGAGTGTGGAT-3′	TagCopenhagen
IL-6 F′	5′-CTGCAAGAGACTTCCATCCAGTT-3′	TagCopenhagen
IL-6 R′	5′-GAAGTAGGGAAGGCCGTGG-3′	TagCopenhagen
IL-10 F′	5′-AGGACTTTAAGGGTTACT-3′	TagCopenhagen
IL-10 R′	5′-AATGCTCCTTGATTTCTG-3′	TagCopenhagen
TNF F′	5′-TGGCCTCCCTCTCATCAGTTC-3′	TagCopenhagen
TNF R′	5′-CCACTTGGTGGTTTGCTACGA-3′	TagCopenhagen
TNF probe	5′-TGGCCCAGACCCTCACACTCAGATCATC-3′	TagCopenhagen

*Primers used for qPCR*.

#### Data Analyses

Analyses performed on digital images were carried out on unmanipulated pictures (OHSC, infarct volume estimations, immunofluorescence and evaluation of mouse cerebrovascular anatomy). On the presented pictures the contrast has been enhanced to allow readers to appreciate the details on small-scale figures. In addition, a histology artifact has been covered in Figure 4A and arteries have been highlighted on zoom in pictures in Figure 4B.

Quantitative data are presented as means ± *SD* and a significant difference was accepted for *p* ≤ 0.05. OHSC data, qPCR data, neurodeficit score, MCA territory, vertical rope test and cell counting analyses were performed using a repeated measure 2-way ANOVA, and PcomA comparisons, infarct comparisons and Western blot densitometry by a non-pared *t*-test. All statistical analyses were followed by an appropriate *post hoc* test and performed using Prism 5 (GraphPad Software, Inc., CA, USA).

## Results

### BID-Deficient Organotypic Hippocampal Slice Cultures (OHSCs) are Partially Protected from OGD-Induced Neuronal Injury

**Figure 1:**
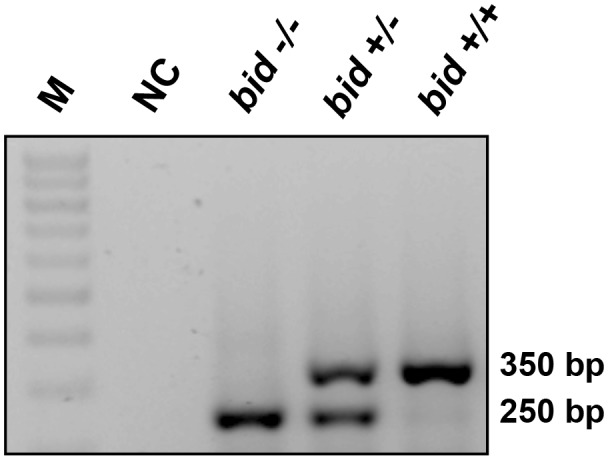
**BID deletion in the applied knockout (KO) strain**. Representative PCR amplification of genomic DNA at the deleted *bid* gene locus by *bid* genotyping reveals the appropriate PCR bands. M: 100 base pair ladder; NC, negative control without the tail DNA template.

PCR-based genotyping confirmed gene deletion in BID-KO mice (Figure 1). To explore whether BID deficiency affords neuroprotection against ischemic neuronal injury in a controlled *in vitro* environment, we initially performed experiments using OHSCs subjected to OGD. OGD is a widely used *in vitro* model of ischemic injury that produces excitotoxic and non-excitotoxic, apoptotic and necrotic neuronal injury (Goldberg and Choi, 1993; Gwag et al., 1995; Kalda et al., 1998).

**Figure 2:**
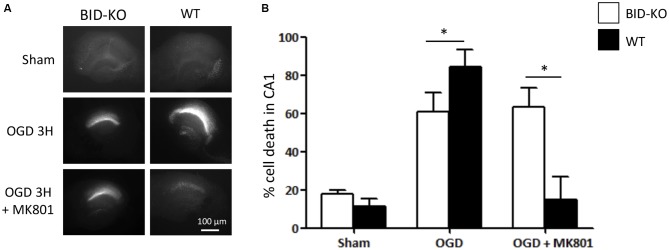
**Organotypic hippocampal slice cultures (OHSCs) isolated from BID deficient mice show protection in response to oxygen-glucose deprivation (OGD).** The slices were sham-exposed or subjected to OGD conditions for 180 min in the absence or the presence of 10 μM MK-801 and allowed to recover for 24 h. **(A)** Representative images of OHSCs derived from BID-KO and WT mice. **(B)** Quantification of injury assessed by PI staining 24 h post-treatment in the CA1 subfield of the hippocampal slices. In the absence of MK-801, BID deficiency protected against neuronal injury (*p = 0.024). Note the protection exerted by MK-801 against OGD-induced neuronal injury of WT OHSCs. In BID-KO slice cultures MK-801 failed to reduce OGD-induced neuronal injury. Scale bar: 100 μm, (*n* = 3–7 in each group, 2-way ANOVA, mean ± *SD*).

OHSCs derived from WT and BID-KO mice were subjected to OGD for 180 min in the presence or absence of the NMDA receptor antagonist, MK-801, a treatment that blocks the excitotoxic component of OGD-induced neuronal injury (Goldberg and Choi, 1993). OHSCs slices were allowed to recover under normoxic and normal glucose conditions over a 24 h time period, after which neuronal injury was determined by PI uptake (Figure 2A). Quantification of PI uptake demonstrated that BID deficiency resulted in a significant neuroprotection in the CA1 subfield if OGD was conducted in the absence of MK-801 (*p* = 0.024). In WT OHSCs, MK-801 exerted potent protection against OGD-induced neuronal injury as expected (Figure 2B). Interestingly, MK-801 failed to further reduce OGD-induced neuronal injury in BID-KO slice cultures, suggesting that BID also mediated neuroprotective signaling during OGD.

### Cerebrovascular Anatomy of BID-KO Mice

**Figure 3:**
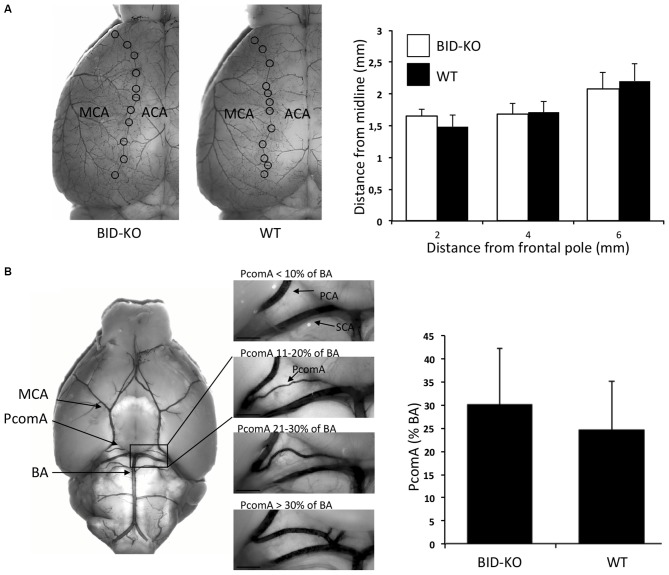
**Cerebrovascular anatomy. (A)** The territory of the middle cerebral artery (MCA) was measured as the distance from the line of anastomoses between the MCA and the anterior cerebral artery (ACA) to the midline at 2, 4 and 6 mm from the frontal pole. The cerebrovascular anatomy was analyzed by visualizing the arteries with cresyl violet solution. We observed no significant difference between BID-KO mice and WT mice at any of these three sites (*p* = 0.96, *n* = 10–13 in each group, 2-way ANOVA mean ± *SD*). **(B)** The diameters of the posterior communicating arteries (PcomAs) were measured at the widest part and the values were calculated as a percentage of the basilar artery (BA) diameter. We found no significant difference between the two groups of mice (*p* = 0.11, *n* = 10–13 in each group, non-paired *t*-test, mean ± *SD*). SCA: Superior cerebellar artery.

We next explored the role of BID in the modulation of ischemic neuronal injury *in vivo*. In order to be able to draw valid conclusions from infarct volumetric analysis of BID-KO and WT mice, we compared the cerebral vascular anatomy in these mice by analyzing the MCA territory and the PcomAs (Figure 3). Comparison of the MCA territory, by measuring the distance from the peripheral anastomoses between the ACA and the MCA at 2, 4 and 6 mm from the frontal pole, revealed no differences between BID-KO and WT mice (*p* = 0.96; Figure 3A). The PcomAs influence the collateral flow to the territory distal to an occluded MCA (Carmichael, 2005) and the size of the PcomAs is a risk factor for ischemic infarction (Schomer et al., 1994). Even though we observed great variations in the development of the PcomAs in individual mice, there was no significant difference in the size of the PcomAs as a percentage of the diameter of the BA between the BID-KO and WT mice (*p* = 0.11; Figure 3B). These findings suggest that the cerebrovascular anatomy in BID-KO and WT mice is comparable.

### BID Deficiency did not Affect Infarct Size or Functional Outcome after tMCAO

**Figure 4:**
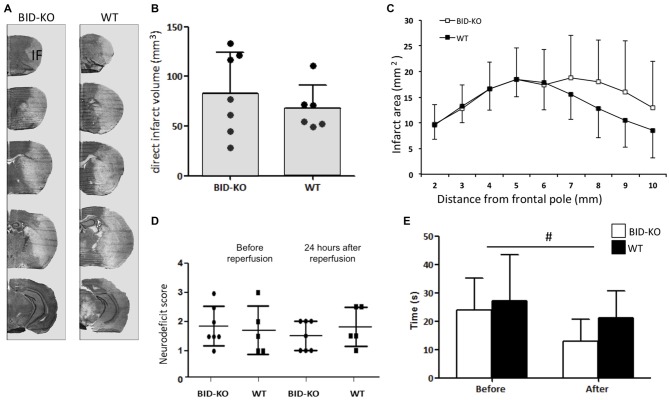
**BID deficiency does not affect infarct formation or improves functional outcome after tMCAO**. **(A)** Representative cresyl violet stained coronal sections of brains from BID-KO mice and WT mice. IF: Infarct. **(B)** Direct infarct volume estimations showed no significant difference in infarct volume between BID-KO mice and WT mice (*p* = 0.45, non-paired *t*-test). **(C)** This was also true for the rostrocaudale distribution analyses (*p* = 0.45, *n* = 6–7 in each group, mean ± *SD*). **(D)** To evaluate the neurodeficit of mice we applied a scoring system from 0–4. We did not see a difference either before or after reperfusion (*p* = 0.73), just as no difference was observed between BID-KO mice and WT mice at any of the two time points (*p* = 0.52, *n* = 6–7 in each group, 2-way ANOVA, mean ± *SD*). **(E)** Mice spent significantly less time on the vertical rope 24 h following reperfusion compared to before surgery (^#^*p* = 0.013), however, there was no difference between the two groups of mice at any of the time points (*p* = 0.29, *n* = 6–7 in each group, 2-way ANOVA, mean ± *SD*).

Infarct volume estimations were determined on the basis of cresyl violet stained brain sections (Figure 4A). Comparison of the mean infarct volume between BID-KO mice and WT mice showed that BID deficiency did not affect infarct size 24 h after 60 min of MCAO (*p* = 0.45; Figure 4B). This was still valid when infarct size was corrected for hemispheric swelling (indirect infarct volume) and different brain sizes (data not shown). The analysis of the rostrocaudal infarct distribution on individual sections also showed no indication of a protective role of BID deficiency after tMCAO (Figure 4C).

The behavioral outcome after tMCAO was evaluated using a neurological deficit score and a vertical rope test. The neurological deficit score was determined after surgery before reperfusion, and again 24 h after reperfusion. We observed no difference in the deficit mean score either before or after reperfusion (*p* = 0.73). Also no difference was detected between BID-KO mice and WT mice at any of the two time points investigated (*p* = 0.52; Figure 4D). Using the vertical rope test, we found that mice spent significantly less time on the rope 24 h after reperfusion compared to before surgery (*p* = 0.013), however, there was no significant difference between the two groups of mice (*p* = 0.29; Figure 4E).

**Figure 5:**
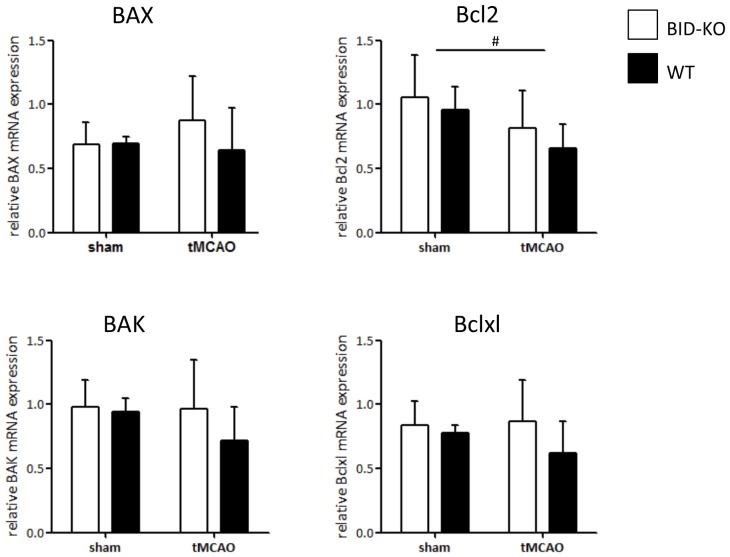
**mRNA expression of downstream regulators of mitochondrial mediated apoptosis**. The mRNA level of BAX, BAK, Bcl2 and Bclxl was examined by qPCR on whole brain lysates and compared between BID-KO mice and WT mice exposed to tMCAO or sham surgery. We found no significant difference between BID-KO mice and WT mice for any of the examined mitochondrial apoptosis regulator genes. Overall the direction of expression was however similar for all four examined genes after tMCAO with the highest expression level found in BID-KO mice (BAK: *p* = 0.34, BAX: *p* = 0.44, Bcl2: 0.33, Bclxl: *p* = 0.25, *n* = 3 in each sham group, *n* = 5–6 in each tMCAO group, mean ± *SD*). There was a significantly lower Bcl2 expression level in tMCAO mice compared to sham mice (^#^*p* = 0.047), but not for BAX, BAK or Bclxl (*p* = 0.65, *p* = 0.42, *p* = 0.63 respectively, *n* = 3 in each sham group, *n* = 5–6 in each tMCAO group, non-paired *t*-test, mean ± *SD*).

To examine whether the lack of effect on infarct size and functional outcome was caused by altered expression level of downstream apoptotic regulators in BID-KO mice, the mRNA level of proapoptotic BAX and BAK, and antiapoptotic Bcl2 and Bclxl were analyzed by qPCR. However, we did not detect any difference in expression level between the two groups of mice for any of the examined apoptotic regulators (*p* = 0.34 (BAK), *p* = 0.44 (BAX), *p* = 0.33 (Bcl2), *p* = 0.25 (Bclxl); Figure 5). We observed, however, reduced levels of Bcl2 mRNA in infarcted vs. sham tissue, suggesting that the mitochondrial apoptosis pathways were activated by cerebral ischemia (Figure 5).

### Microglial/Leukocyte Response to tMCAO

**Figure 6:**
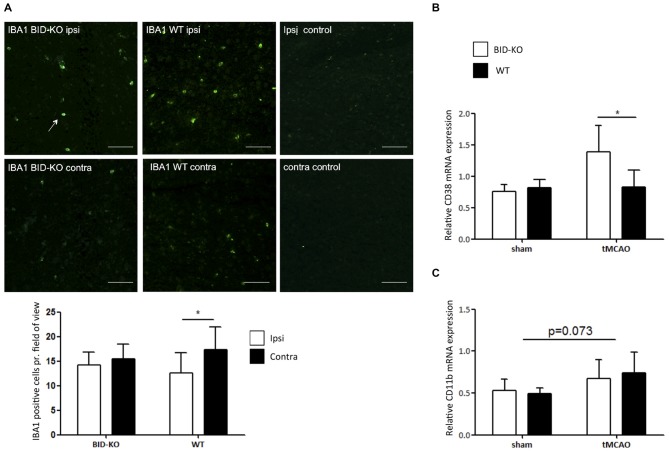
**Microglial/leukocyte response after transient middle cerebral artery occlusion (tMCAO)**. **(A)** IBA1 staining of ipsilateral and contralateral hemisphere of one representative BID-KO and WT mouse exposed to tMCAO. We observed no significant difference in the number of IBA1^+^ cells per field of view between BID-KO mice and WT mice exposed to tMCAO on the contralateral hemisphere or the ipsilateral hemisphere (*p* = 0.93), however, in the brains of WT mice, the expression was significantly higher in the ipsilateral hemisphere compared to the contralateral hemisphere (*p = 0.017). This difference was not observed in the brains of BID-KO mice. The white arrow marks an IBA1 positive cell. The pictures were taken from normal appearing cortex. Scale bars: 60 μm (*n* = 5–6 in each group, 2-way ANOVA, mean ± *SD*). **(B)** The relative mRNA expression of CD38 was significantly higher in BID-KO mice compared to WT mice exposed to tMCAO 24 h after surgery (*p = 0.05, *n* = 3 in each sham group, *n* = 5–6 in each tMCAO group, 2-way ANOVA, mean ± *SD*). **(C)** The relative mRNA expression level of CD11b was not quite upregulated 24 h after surgery (*p* = 0.073), and there was no significant difference between BID-KO and WT sham mice or between BID-KO and WT tMCAO mice (*p* = 0.87, *n* = 3 in each sham group, *n* = 5–6 in each tMCAO group, 2-way ANOVA, mean ± *SD*).

IBA1 is upregulated in microglia/leukocytes in response to focal cerebral ischemia (Ito et al., 2001). In this study, estimation of the number of IBA1^+^ microglia/leukocytes was performed and compared between the ipsilateral hemisphere and the contralateral hemisphere of mice exposed to tMCAO. We observed no significant difference in the number of IBA1^+^ cells between BID-KO mice and WT mice on the contralateral hemisphere or the ipsilateral hemisphere (*p* = 0.93). When comparing the number of IBA1^+^ cells between the hemispheres within the same group of mice, we found that there were significantly more IBA1^+^ cells in the ipsilateral hemisphere of WT mice compared to the contralateral hemisphere (*p* = 0.017). This significant difference was not observed in the BID-KO mice, suggesting altered activation or recruitment of microglia/leukocytes in BID-KO mice in response to focal cerebral ischemia (Figure 6A).

CD38 is a surface protein expressed on various immune cells. It is considered to be a marker of activation known to hold various immune regulatory functions (Quarona et al., 2013). To investigate the immune cell response further, qPCR for CD38 was performed on whole brain mRNA extracts. In this analysis, we observed a significantly higher level of CD38 in BID-KO mice compared to WT mice after tMCAO (*p* = 0.05), whereas levels were similar in mice exposed to sham surgery (Figure 6B). We also examined the mRNA level of the microglia/leukocyte marker CD11b. Here, we did not find a significant difference between BID-KO mice and WT mice (*p* = 0.87; Figure 6C).

### Astroglial Response to tMCAO

**Figure 7:**
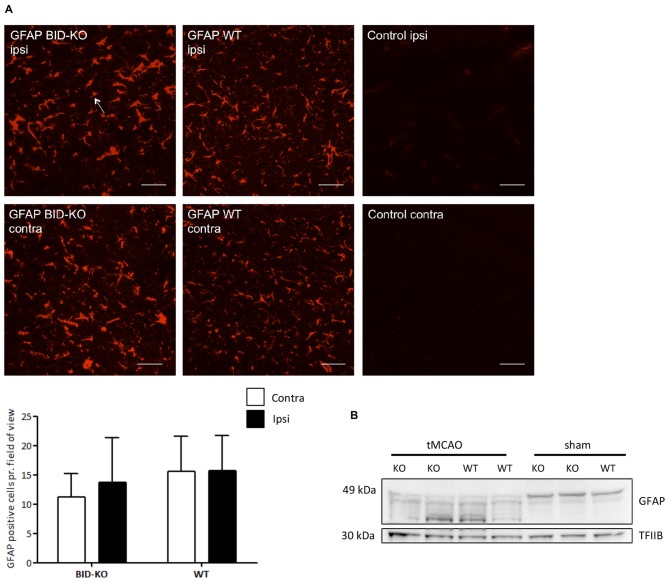
**Astrocyte response after transient middle cerebral artery occlusion (tMCAO).** **(A)** When we compared the number of GFAP positive cells per field of view between BID-KO mice and WT mice exposed to tMCAO, we found no difference between the ipsilateral and the contralateral hemisphere of either of the two groups of mice (*p* = 0.52) or between the two groups of mice at any of the hemispheres (*p* = 0.23). The white arrow marks a GFAP positive cell. The pictures were taken in the normal appearing cortex. Scale bars: 30 μm (*n* = 5–6 in each group, 2-way ANOVA, mean ± *SD*). **(B)** Western blot performed on whole brain lysate showed an activation of GFAP in both BID-KO mice and WT mice exposed to tMCAO as indicated by the expression pattern of the various isotypes of GFAP compared to sham mice.

Immunohistochemistry for GFAP was performed, and an estimation of the number of GFAP^+^ astrocytes pr. field of view was carried out and compared between the ipsilateral hemisphere and the contralateral hemisphere of mice exposed to tMCAO. We detected no difference in the number of GFAP^+^ cells between the two hemispheres (*p* = 0.52) or between BID-KO and WT mice (*p* = 0.23; Figure 7A).

In addition to immunohistochemistry, Western blotting for GFAP was performed on whole brain lysates from both tMCAO and sham mice. We observed astroglial activation in both BID-KO and WT mice subjected to tMCAO, as evidenced by the GFAP expression pattern revealing isotypes known to be expressed by activated astrocytes (Kamphuis et al., 2012). These were not present in the sham-operated mice (Figure 7B). In conclusion, our result suggests that BID deficiency does not affect astroglial activation in response to tMCAO.

### Expression of Inflammatory Markers

**Figure 8:**
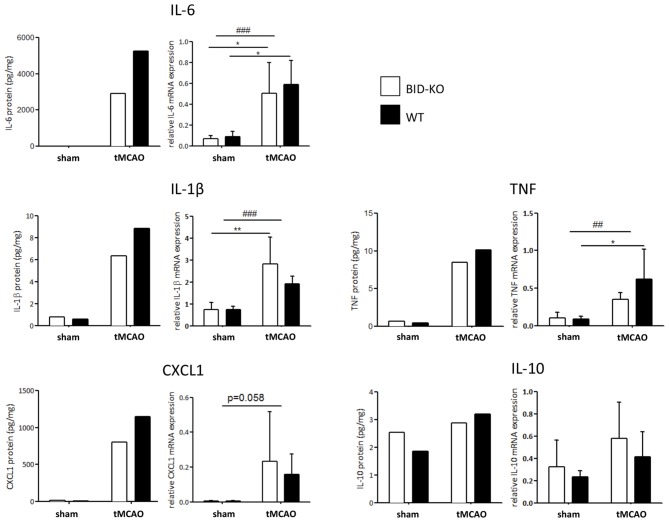
**Expression of cytokines and chemokines.** The expression level of various cytokines and chemokines were evaluated on protein level by mesoscale multiplex analysis of pooled samples (pool from 3 mice in each sham group, and 5–6 mice in each tMCAO group), and on mRNA by qPCR of non-pooled samples. As expected, the expression was generally elevated after tMCAO on both protein and mRNA level (*p < 0.05, **p < 0.01, ^##^*p* < 0.01, ^###^*p* < 0.001). When comparing the expression between the two groups of mice after tMCAO there was an overall reduction in protein levels in the BID-KO mice compared to WT mice. The biggest reduction of expression in BID-KO mice was found for IL-6 which was reduced by ~45%. For CXCL1 protein the expression was reduced by ~30%, IL-1β protein by ~28%, TNF protein by ~16% and IL-10 protein by ~9%. However, all cytokines and chemokines were not reduced in BID-KO mice compared to WT mice on mRNA level, and there was no significant difference for any of the examined cytokines and chemokines (IL-6: *p* = 0.65, IL-1β: *p* = 0.27, CXCL1: *p* = 0.7, TNF: *p* = 0.33, IL-10: *p* = 0.33, 2-way ANOVA, mean ± *SD*).

The expression level of various cytokines and chemokines were analyzed on protein level by mesoscale multiplex analysis of pooled protein samples. As expected, we observed an increase in expression level of the examined cytokines in response to tMCAO. As this analysis was carried out with pooled samples, statistics cannot be performed, but there was a clear tendency that the examined cytokines and chemokines were reduced in BID-KO mice exposed to tMCAO compared to WT mice. IL-6 levels were found to be reduced by ~45%, CXCL1 by ~30%, IL-1β by ~28% and TNF by ~16%. In the case of IL-10 protein, we noted no considerable difference between the groups (~9% lower in BID-KO mice; Figure 8). The expression level of the cytokines and chemokines were in addition analyzed on mRNA level by qPCR. Here we did not find a similar expression pattern, and there was no significant difference between BID-KO mice and WT mice for any of the examined cytokines and chemokines (Figure 8).

**Figure 9:**
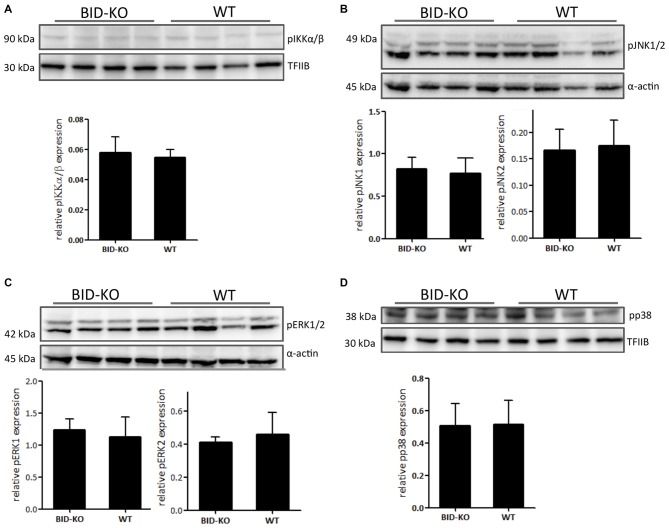
**Expression of phosphorylated signaling pathway components.** **(A)** The level of phosphorylated NF-κB upstream kinase IKKα/β was compared by Western blotting and subsequent densitometry between BID-KO mice and WT mice on whole brain lysates 24 h after tMCAO. We found no difference between the two groups of mice (*p* = 0.63). **(B)** This was also true for the phosphorylated MAP kinases JNK1/2 (pJNK1: *p* = 0.69, pJNK2: *p* = 0.78), **(C)** ERK1/2 (pERK1: *p* = 0.57, pERK2: *p* = 0.5), and **(D)** p38 (*p* = 0.93). Probing for α-actin or TFIIB served as loading control (*n* = 4 in each group, non-paired *t*-test, mean ± *SD*).

The MAPK and NF-κB signaling cascades are known to be important in innate immunity and inflammation (Chen et al., 2001; Kyriakis and Avruch, 2012) and they have been shown to be downregulated in BID deficient compared to WT cell cultures following inflammatory stimuli exposure (Yeretssian et al., 2011). We examined the expression of the phosphorylated MAPKs pERK1/2, pJNK1/2 and pp38, and the NF-κB signaling pathway component pIKKα/β by Western blotting performed on protein extractions from whole brain lysate. The obtained results in these analyses did not reveal a difference between the two groups (Figure 9).

## Discussion

The present study demonstrated a neuroprotective effect of BID deficiency against OGD-induced neuronal injury *in vitro*, yet we did not find a significant effect of BID deficiency on lesion volume or functional outcome in response to tMCAO *in vivo*. We also examined the effect of BID deficiency on the inflammatory responses to focal ischemia *in vivo*. We found that BID modulated inflammation after tMCAO; Microglial/leukocyte response was reduced in BID-KO mice as indicated by changes in IBA1 and CD38 expression, and the overall level of inflammatory cytokines and chemokine protein was reduced in BID-KO mice compared to WT mice.

Apoptosis contributes significantly to cell death after stroke (Mergenthaler et al., 2004; Lorz and Mehmet, 2009). It is well established that BID is a proapoptotic protein (Wang et al., 1996; Chung et al., 2010) and two previously published studies using a different genetic strain have shown that BID-KO mice developed smaller infarcts than WT mice (Plesnila et al., 2001; Yin et al., 2002). Using well-characterized BID-deficient mice (Kaufmann et al., 2007; König et al., 2014) and after careful evaluation of their cerebrovascular anatomy, we did not find any difference in infarct volume between BID-KO and WT mice after 60 min of transient focal cerebral ischemia. The mean infarct volume of BID-KO mice actually tended to be higher than the mean infarct volume of WT mice (83.26 and 68.28 mm^3^ respectively). In addition, we did not find any differences in the functional outcome between BID-KO mice and WT mice. A recent study from our laboratory demonstrated a robust protection against tMCAO induced neuronal injury as a result of BAX gene deletion (D’Orsi et al., 2015). As BID is acting upstream of BAX in cell death signaling, these results indicate that other upstream, proapoptotic BH3-domain containing BCL-2 family proteins (“BH3-only proteins”) such as BIM and PUMA may play a more predominant role in BAX activation after cerebral ischemia (Ren et al., 2010; Engel et al., 2011). A second interesting observation in this regard came from our *in vitro* studies in OHSCs. Here, exposure to the NMDA receptor antagonist MK-801 afforded robust protection against OGD-induced neuronal injury in WT cultures, but failed to provide protection in BID-KO cultures above that induced by BID deficiency. It is therefore possible that BID modulates neuroprotective signaling during excitotoxic injury. For example, inflammatory cytokines (including IL-1β and IL-6) have previously been shown to be protective against neuronal injury induced by NMDA receptor overactivation (Carlson et al., 1999). A reduction in IL-1β and IL-6 levels in BID-KO animals may impair endogenous neuroprotective signaling through these cytokines. It is therefore possible that any neuroprotection afforded by reduced apoptotic signaling through the mitochondrial apoptosis pathway in the *bid*-deficient animals is cancelled out by an inhibition og endogenous, pro-inflammatory signals that promote neuroprotection.

BID-KO mice used in previous studies that have shown an infarct reducing effect of BID deficiency originated from a different mouse strain (Yin et al., 1999). This BID-KO strain was generated on a mixed C57BL6/Sv129 genetic background and the mice genome still contained the drug selection cassette from the targeted DNA locus (Yin et al., 1999). The mice strain employed in the present study was generated in the Andreas Strasser group on a pure C57BL/6 background, and the drug selection cassette was removed by breeding with Cre-positive “deleter” females (Kaufmann et al., 2007). The difference between our and previous studies could be a result of strain differences. It has also been suggested that drug selection cassettes left in the genome could affect expression of linked genes (Koyasu et al., 1994). Interestingly, phenotypical differences between the two different BID-KO mice strains have previously been described: The mice utilized in the two previous studies have been observed to develop spontaneous myeloid leukemia as they age (Zinkel et al., 2003). This was not observed for the mice in the present study (Kaufmann et al., 2007). It should also be noted that the models used in the two studies were slightly different from the model applied in the present study. In the study by Plesnila et al. (2001), mice were exposed to 30 min of ischemia and 48 h of survival applying a model allowing only unilateral carotid artery reperfusion. In the study by Yin et al. (2002), mice were exposed to 90 min of ischemia and 72 h of survival, applying a model allowing bilateral carotid artery reperfusion. The differences in a potential BID involvement in ischemic neuronal injury may therefore be a consequence of differences in excitotoxic and/or inflammatory signaling between the different models employed.

As it has previously been shown that BID affects the inflammatory response both *in vitro*, and *in vivo* (Scatizzi et al., 2007; Chung et al., 2010; Mayo et al., 2011; Yeretssian et al., 2011; König et al., 2014), we therefore chose to look at microglial/leukocyte activation in our model. Microglial cells are known to contribute substantially to the elevation of cytokine levels in the brain after focal cerebral ischemia (Lambertsen et al., 2012) and the lack of IBA1 microglial/leukocyte activation or recruitment in the ipsilateral hemisphere in BID-KO mice observed in the present study may be responsible for a lower levels of proinflammatory cytokines in BID-KO mice. However, it should be mentioned that the increase in IBA1 positive cells observed in WT mice was modest and statistical analysis between the ipsilateral hemisphere of BID-KO mice and WT mice did not reveal a significant difference. The elevation of CD38 mRNA, which is mainly expressed by infiltrating cells and has been found to contribute to tissue damage after focal cerebral ischemia (Choe et al., 2011), and the lack of reduction in mRNA levels of inflammatory cytokines and chemokines in BID-KO mice exposed to tMCAO are at first glance conflicting observations in this regard, and might be a result of unidentified compensatory mechanisms. We do not see an elevation of CD38 or CD11b mRNA at this time point in WT mice. This is however not an unexpected finding as infiltration of cells is known to be sparse 24 h after tMCAO (Schilling et al., 2003).

Based on the data presented here, we suggest that the general perception of BID as an important player of cell death after focal cerebral ischemia should be interpreted with caution. While we observed partial protection in an *in vitro* model of ischemic neuronal injury in OHSC, we did not find evidence for a robust protection against tMCAO *in vivo*. The modulation of the inflammatory response observed for BID-KO mice in the present study was not sufficient to influence infarct volume or acute neurological scores in response to tMCAO. BID may still be an interesting subject in the stroke context, as we here show that BID modulates the inflammatory response to tMCAO. Indeed, it is well established that the inflammatory response plays an important role in the pathophysiology of recovery and regeneration after stroke (Lambertsen et al., 2012).

## Author Contributions

NAM conducted animal surgeries, performed experiments, interpreted results, performed statistical analysis and drafted the manuscript. GC conducted animal surgeries, performed experiments and interpreted results. HGK, MLE, HB, BD, SP performed experiments and interpreted results. MS and TD performed multiplex analyses. JHP and KLL conceived the study, interpreted results and helped draft the manuscript. All authors red and approved the final manuscript.

## Conflict of Interest Statement

The authors declare that the research was conducted in the absence of any commercial or financial relationships that could be construed as a potential conflict of interest.

## Abbreviations

ACA: Anterior cerebral artery
BA: Basilar artery
BCL-2: B-cell lymphoma-2
BID: BH3 interacting-domain death agonist
CCD: Charge-coupled device
CCL-2: Chemokine (C-C motif) ligand 2
CXCL1: Chemokine (C-X-C motif) ligand 1
ECL: Enhanced chemiluminescence
ERK: Extracellular signal-regulated kinase
GAPDH: Glyceraldehyde 3-phosphate dehydrogenase
GFAP: Glial fibrillary acidic protein
HPRT1: Hypoxanthine phosphoribosyltransferase 1
HRP: Horse radish peroxidase
IBA1: Ionized calcium-binding adaptor molecule 1
IFNγ: Interferon-γ
IL: Interleukin
KO: Knockout
MCA: Middle cerebral artery
NF-κB: Nuclear factor κB
NOD: nucleotide-binding oligomerization domain containing
OGD: Oxygen-glucose deprivation
OHSC: organotypic hippocampal slice cultures
ON: over night
PFA: Paraformaldehyde
qPCR: Quantitative PCR
PcomA: Posterior communicating artery
PVDF: Polyvinylidene difluoride
rCBF: Regional cerebral blood flow
RT: Room temperature
SCA: Superior cerebellar artery
tBID: truncated BID
TFIIB: Transcription initiation factor IIB
tMCAO: Transient middle cerebral artery occlusion
TNF: Tumor necrosis factor
WT: Wild-type
γTri-DAP: L-Ala-γ-D-Glu-*meso*-diaminopimelic acid.
